# Grain yield and interspecific competition in an oat-common vetch intercropping system at varying sowing density

**DOI:** 10.3389/fpls.2024.1344110

**Published:** 2024-03-08

**Authors:** Ying Wang, Xue Han, Xingyao Zhao, Yanli Zhang, Bingjie Qi, Lijun Li

**Affiliations:** College of Agronomy, Inner Mongolia Agricultural University, Hohhot, China

**Keywords:** legume-cereal intercropping, land equivalent ratio, economic efficiency, competition indices, sowing density

## Abstract

**Introduction:**

Oat (*Avena nuda* L.) and common vetch (*Vicia sativa* L.) intercropping in the northern regions of China has resulted in substantial production capabilities. However, there is currently a dearth of comprehensive research on whether this intercropping system can enhance productivity through increased sowing densities and underlying interspecies interaction mechanisms.

**Methods:**

A two-year field experiment was conducted in 2022 and 2023 to investigate the yield, biological efficiency, economic efficiency, and competition indicators of oats and common vetch in a high-density intercropping system. Two cropping patterns (monocropping and intercropping) and five sowing densities (D1: 4.5×10^6^ plants ha^-1^; D2:5.4×10^6^ plants ha^-1^; D3:6.3×10^6^ plants ha^-1^; D4: 7.2×10^6^ plants ha^-1^; and D5: 8.1×10^6^ plants ha^-1^) were arranged in a randomized block design.

**Results:**

At the same sowing density, the intercropped oats exhibited greater grain yield than the monocultures. Increasing the oat sowing density significantly enhanced oat yield, with the D3 level in intercropping showing the highest yield increase, ranging from 30.98% to 31.85%, compared with the monoculture. The common vetch intercropping grain yield was maximized in the D2 treatment. The land equivalent ratio was maximized at the D2 level in both years and was significantly higher than D1, with the land equivalent coefficient, system productivity index, and percentage yield difference suggesting that increasing oat sowing densities improved the productivity of the intercropping system, with the best performance observed at the D2 level. For both years, the proportionate actual yield loss of oat was the highest at the D3 level; significantly surpassing D1, proportionate actual yield loss of common vetch and actual yield loss were the highest at level D2, both significantly surpassing D1. These indicates that appropriate densification contributes to the realization of the advantages of intercropping. With an increased oat sowing density, the economic benefits of the intercropping system were maximized at the D2 and D3 levels. Regarding intercropping competition, oat was the dominant crop under different sowing densities (Aggressivity for oat (A_O_)>0, relative crowding coefficient for oat (K_O_)>1, competition ratio for oat (CR_O_)>1), whereas common vetch was the inferior crop. Compared with the D1 level, the D2 level harmonized the aggressivity, competitive ratio, and relative crowding coefficients of oat and common vetch, significantly increasing crowding coefficient for common vetch (K_V_) and competition ratio for common vetch by 19.76% to 21.94% and 4.80% to 7.51%, respectively, while reducing K_O_ and CR_O_.

**Discussion:**

This result suggests that in the intercropping of common vetch and oat in alpine regions, rational densification can harmonize interspecific competition and thus improve the biological efficiency and economic benefits of intercropping systems.

## 1 Introduction

The limited expansion of arable land poses a significant impediment to the augmentation of total grain production in China, and enhancing the productivity per unit of land remains an ongoing challenge (Han et al., 2022). Intercropping can establish a stratified and distinct crop canopy structure above the ground, intercept more light energy, improve ventilation and light transmission conditions and take advantage of the side rows (Gong et al., 2020). Cereal-legumes can improve the soil nutrient microenvironment through nitrogen fixation of belowground legume rhizomes, and significantly improve the productivity of the land per unit area, which has become an important strategy for organic and smallholder agriculture (Maingi et al., 2001; Li et al., 2003; Yu et al., 2015). Intercropping common vetches with oats has been widely used for forage production in alpine regions such as the Tibetan Plateau and along the foothills of the Yinshan Mountains (Lithourgidis et al., 2006). This production model can be better adapted to the arid and cold environments in northern China, guaranteeing the production of high-quality green forage in alpine pasture areas (Wang et al., 2020). Recent studies have suggested that intercropping systems effectively address challenges in common vetch, such as weak stems and poor erect growth, while providing structural support for common vetch clover growth and facilitating the mechanical harvesting of legume seeds (Lithourgidis et al., 2006; Nguyen et al., 2020). These studies have provided new ideas to solve the problems of low grain yield and high harvest loss of common vetch; however, less attention has been currently paid to the seed production capacity of the oat intercropping common vetch model.

Increasing the sowing density is an effective measure to increase crop yield, but it is often accompanied by higher water and nitrogen demands within the crop population, resulting in increased competition for both aboveground (light) and below-ground (water and nutrients) resources (Zhai et al., 2018). Owing to interspecific spatial and temporal complementarities, intercropping has a higher resource use efficiency than monocropping, which helps optimize the sowing density of combined crops to maximize the yield benefits of intercropping systems (Willey and Osiru, 1972). Previous studies have shown that increasing the density of maize can improve the photosynthetic capacity of intercropping systems, improve the microstructure of leaves (Yang et al., 2022), increase the nitrogen use efficiency of intercropping systems (Xu et al., 2021), and thus increase the total yield of intercropping systems (Fan et al., 2019). As sowing density increase, the dry matter partitioning into the leaves and stems of crops increases, as increased competition for light, water, and nutrients may also partition too much dry matter into the roots (Hauggaaed-Nielsen et al., 2006; Freschet et al., 2018; Zhang et al., 2020). For Poaceae crops, an excessively high sowing density could lead to the development of ineffective tillers, wasteful nutrient and water utilization, and premature senescence owing to shading, all of which are detrimental to high yields (Jiang et al., 2023). However, few studies on density tolerance in intercropping Poaceae crops have been reported. Therefore, further research is necessary to determine whether increasing the density of intercropped oats in oat and common vetch intercropping systems can enhance the overall productivity.

The main reason for intercropping to increase yield is to provide a basis for ecological niche separation for crops with different demand characteristics, which contributes to competition and complementarity between crops and improves resource utilization; that is, crop interspecific relationships are important factors affecting the yield of intercropping. Intercropping systems in a certain range of interspecific competitiveness tend to be enhanced with an increase in the density of a particular crop, which is an important breakthrough point to increase competitive advantage. Further optimization of interspecific relationships is the ecological basis for improving the yield advantages of intercropping (Hauggaaed-Nielsen et al., 2006; Abdul Rahman et al., 2021). According to the study of Mead and Willey (1980), any attempt to explain intercropping advantages using a single index may be problematic, and different aspects of intercropping data should be explained by different indices. The current indicators for evaluating intercropping systems mainly include biological, economic efficiency and interspecific relationships, which have been applied to evaluate potatoes intercropped with legumes (Gitari et al., 2020) and peppermint intercropped with soybean (Machiani et al., 2018) and provide a more comprehensive evaluation of intercropping interspecific relationships. The application of these indicators to evaluate the effect of the sowing density of oats on the grain yield of the common vetch intercropping system has positive significance in improving the intercropping productivity of this model.

We hypothesized that oats intercropped with common vetch would exhibit better tolerance to planting density compared to sole cropping, and that different oat sowing densities would alter the interspecific relationship. This study aimed to (1) assess the grain production performance of oats intercropped with common vetches under various oat sowing densities and (2) elucidate the changes in interspecific competition intensity between the two crops under different oat sowing densities. The findings of this study can serve as evidence in the context of inter-specific competition, thereby contributing to the enrichment of forage planting models and the judicious densification of intercropping systems involving Poaceae crops.

## 2 Materials and methods

### 2.1 Experimental site

**Figure 1:**
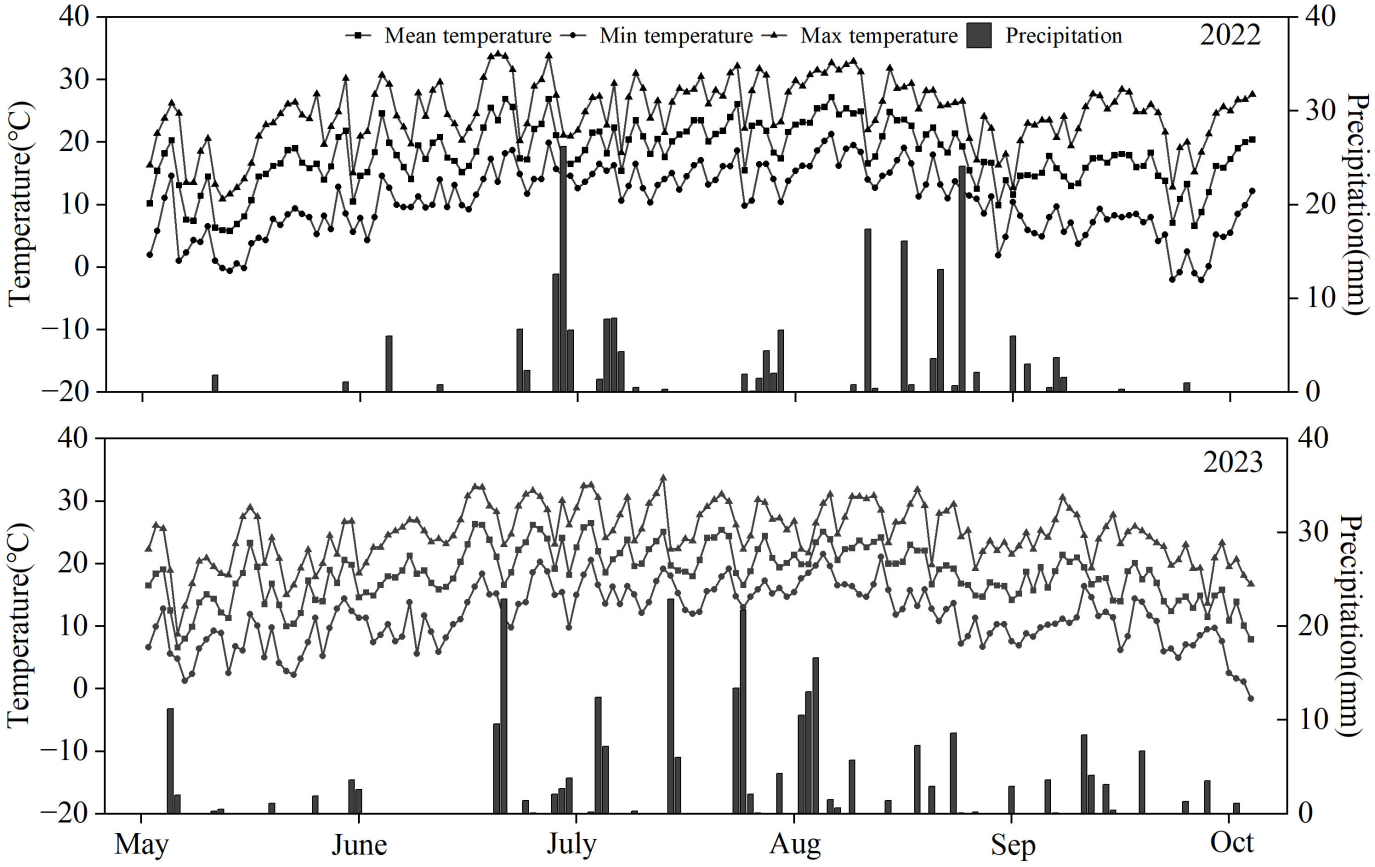
Changes of daily maximum temperature, daily minimum temperature, daily mean temperature and precipitation at the study site in 2022~2023.

This study was conducted from 2022 to 2023 at the Experimental Station of the Institute of Agricultural and Animal Husbandry Sciences in Ulanqab. The Station is located in Pingdiquan Town (40.92°N, 113.11°E), with an average altitude of 1,417 m, in the Chahar Right Front Banner, Ulanqab City, Inner Mongolia Autonomous Region, which is located in the northern agricultural and pastoral intertwined area of China. It has a temperate continental monsoon climate, with a long-term average annual temperature of 5.0°C, annual sunshine hours of approximately 3,000, and an annual frost-free period of 120 days. The long-term average annual evapotranspiration is approximately 1,957.7 mm, and the annual precipitation ranges from 340 to 450 mm. The test soil was a calcareous chestnut soil, and the basic nutrients of the soil before sowing were: organic matter of 1.23%, available nitrogen of 86.24 mg kg^-1^, available P of 12.17 mg kg^-1^, available K of 132.18 mg kg^-1^, and pH of 7.68. Precipitation and temperature during the experimental period are shown in **Figure 1**.

### 2.2 Experimental design

The experiment was conducted in a randomized block design with two factors: the first factor was cropping pattern, with two treatments: oat intercropped with common vetch(I) and oat monocropping(S); and the second factor was oat sowing density, with five treatments: The sowing density of oat were 4.50×10^6^ plants ha^-1^ (D1), 5.40×10^6^ plants ha^-1^ (D2), 6.30×10^6^ plants ha^-1^ (D3), 7.20×10^6^ plants ha^-1^ (D4), and 8.10×10^6^ plants ha^-1^ (D5). The intercropped strip received the same sowing densities as that used for monocropping. In addition, common vetch monoculture treatment (SV) was set up, the sowing density of common vetch monocropping was 1.50×10^6^ plants ha^-1^. Different oat sowing density were formed by keeping row spacing constant while changing seed-to-seed distance. In intercropping plots, oat and common vetch were planted in 125 cm wide strips, with 2/5 occupied by oat (a strip 50 cm wide) and the remaining 3/5 occupied by common vetch (a strip 75 cm wide).

In this experiment, both oats and common vetch were sown in wide rows with a spacing of 25 cm and a seed strip width of 8 cm, with a plot area of 25 m^2^ (5.0×6.0 m) for each treatment, and four natural strips were set up in each plot. Before sowing, the experimental plots were ploughed 20~30 cm and irrigated, and 150 kg·ha^-1^ of compound fertilize r (N, 15%; P_2_O_5_, 15%; K_2_O, 15%) was applied to each treatment. Pure nitrogen at 63 kg·ha^-1^ was applied at the tillering stage and the nitrogen fertilizer was urea (N, 46%). Other management measures were the same as those used in the local fields. The test oats (cv. Bayou 14) were provided by the Institute of Agricultural and Animal Husbandry Sciences in Ulanqab, and the common vetch (cv. Ximu 333/A) were provided by the Inner Mongolia Academy of Agricultural and Animal Husbandry Sciences. Both varieties are resistant to drought, cold, and infertile soils, and are commonly used in local production. The oats and common vetch were sown on May 12, 2022, May 13, 2023, and harvested on August 28, 2022, and September 2, 2023.

### 2.3 Measurements and calculation

#### 2.3.1 Grain yield

At the time of maturity of the oats and common vetch in each plot, we selected plants with a uniform growth of 3 m^2^ (intercropping plots of each of the two crops took 3 m^2^). The harvested kernels were air-dried, cleaned, and weighed, and the common vetch pods and oat wheat ears were threshed separately. Grain moisture determination in China is based on 14% moisture as the standard for storing and marketing grains; therefore, oat and common vetch grain yields were determined at this moisture content.

#### 2.3.2 Economic analysis

Economic indicators were applied to evaluate the economic feasibility of integrating oat and common vetch cropping systems. The total variable cost package expanded oat seed (1.0298 US$ kg^-1^), common vetch seed (1.0985 US$ kg^-1^), compound fertilizer (0.3295 US$ kg^-1^), urea (0.2472 US$ kg^-1^), labor (32.9542 US$ ha^-1^), and machinery (61.789 US$ ha^-1^), and the gross income was obtained by considering the Economic yields based on prevailing market prices (0.8239 and 1.0298 US$ kg^-1^ for oats and common vetch, respectively) were calculated.

#### 2.3.3 Evaluation of the performance of intercropping systems

##### 2.3.3.1 Assessment of the biological efficiency of oat-common vetch intercropping system

The land equivalent ratio (LER) as described by Mead and Willey (1980) indicates the relative area of the monocrop that is required to produce an equivalent yield obtainable under intercropping as shown in Equations 1–3:


(1) $$\text{LER}={\text{LER}}_{\text{O}}+{\text{LER}}_{\text{V}}$$

(2) $${\text{LER}}_{\text{O}}=\frac{{\text{Y}}_{\text{IO}}}{{\text{Y}}_{\text{MO}}}$$

(3) $${\text{LER}}_{\text{V}}=\frac{{\text{Y}}_{\text{IV}}}{{\text{Y}}_{\text{MV}}}$$

Where Y_IO_ and Y_IV_ are the yields of oats common vetch in the intercropping system, and Y_MO_ and Y_MV_ are the yields of sole oat and sole common vetch, respectively. when LER>1 indicates intercropping advantage, and when LER<1 indicates intercropping disadvantage (Machiani et al., 2018).

Land equivalent coefficient (LEC) is a product of LER_O_ and LERv and was assessed using Equation 4, this showed that mixtures with stable potentials are superior to their components planted as sole crops on per unit area productivity (Adetiloye et al., 1983), LEC can clearly distinguish between competitive and complementary intercropping combinations than LER.


(4) $$\text{LEC}=\frac{{\text{Y}}_{\text{IO}}}{{\text{Y}}_{\text{MO}}}\times \frac{{\text{Y}}_{\text{IV}}}{{\text{Y}}_{\text{MV}}}$$

The system productivity index (SPI) (Odo, 1991) was used to assess the productivity and stability of intercropping systems. The main advantage of the SPI is that it standardizes the yields of secondary crops (legumes) with those of the main crop Equation 5.


(5) $$\text{SPI}={\text{Y}}_{\text{IO}}+\frac{{\text{Y}}_{\text{MO}}}{{\text{Y}}_{\text{MV}}}\times {\text{Y}}_{\text{IV}}$$

Percentage yield difference (PYD) refers to the percentage yield difference between a monocrop and an intercrop (Afe and Atanda, 2015). It assumes a monoculture crop yield of 100 percent, where the loss of yield from one crop is usually compensated for by an increase in yield from a companion crop (Gitari et al., 2020). PYD was computed as shown in Equation 6.


(6) $$\text{PYD}=100-(\frac{{\text{Y}}_{\text{MO}}-{\text{Y}}_{\text{IO}}}{{\text{Y}}_{\text{MO}}}+\frac{{\text{Y}}_{\text{MV}}-{\text{Y}}_{\text{IV}}}{{\text{Y}}_{\text{MV}}})\times 100$$

##### 2.3.3.2 Evaluation of the economic efficiency of intercropping systems

The economic yields were converted into oat equivalent yield (OEY) (Gitari et al., 2019), OEY was show in Equation 7.


(7) $$\text{OEY}={\text{Y}}_{\text{IO}}+({\text{Y}}_{\text{IV}}\times \frac{{\text{P}}_{\text{V}}}{{\text{P}}_{\text{O}}})$$

Where P_V_ and P_O_ represent the market prices of oat and common vetch, respectively.

The monetary advantage index (MAI) describes the competitive and economic advantages of intercropping over monocropping (Ghosh, 2004). MAI was determined as indicated in Equation 8. The cropping system with the highest MAI is ranked the most profitable.


(8) $$\text{MAI}=(\frac{\text{LER}-1}{\text{LER}})\times \text{VCI}$$

The value of the combined intercrops (VCI) is calculated using the following Equation 9 (Finney, 1990).


(9) $$\text{VCI}=({\text{Y}}_{\text{IO}}\times {\text{P}}_{\text{O}})+({\text{Y}}_{\text{IV}}\times {\text{P}}_{\text{V}})$$

The relative value total (RVT) Equation 10 illustrates the economic value of the cultivated crop compared to the LER, which is more useful for those farmers aiming to derive economic value from intercropping (Alabi and Esobhawan, 2006).


(10) $$\text{RVT}=\frac{\left({\text{Y}}_{\text{IO}}\times {\text{P}}_{\text{O}})+({\text{Y}}_{\text{IV}}\times {\text{P}}_{\text{V}}\right)}{{\text{Y}}_{\text{MO}}\times {\text{P}}_{\text{O}}}$$

Moseley (1994) gives a slightly more complex but better measure of the economic advantages of intercropping or mixed cropping called the Replacement Value of Intercropping (RVI). The RVI is superior to the RVT because it takes into account the variable costs of the production process, i.e. seed, fertilizer etc (Singh et al., 2015). It was calculated following Equation 11.


(11) $$\text{RVI}=\frac{\left({\text{Y}}_{\text{IO}}\times {\text{P}}_{\text{O}})+({\text{Y}}_{\text{IV}}\times {\text{P}}_{\text{V}}\right)}{{\text{Y}}_{\text{MO}}\times {\text{P}}_{\text{O}}-{\text{C}}_{\text{OS}}}$$

Where C_OS_is the variable cost of oat (the main crop) in a pure stand.

##### 2.3.3.3 Competition indices

The relative crowding coefficient (K) was used as a competitive power coefficient to measure the competitive advantage of one crop over another in intercropping Equations 12–14 (Ghosh, 2004).


(12) $$\text{K}={\text{K}}_{\text{O}}\times {\text{K}}_{\text{V}}$$

(13) $${\text{K}}_{\text{O}}=\frac{{\text{Y}}_{\text{IO}}\times {\text{Z}}_{\text{IV}}}{{\text{Y}}_{\text{MO}}-{\text{Y}}_{\text{IO}}}\times {\text{Z}}_{\text{IO}}$$

(14) $${\text{K}}_{\text{V}}=\frac{{\text{Y}}_{\text{IV}}\times {\text{Z}}_{\text{IO}}}{{\text{Y}}_{\text{MV}}-{\text{Y}}_{\text{IV}}}\times {\text{Z}}_{\text{IV}}$$

K_O_ and K_V_ denote the relative crowding coefficients of oats and common vetches, respectively, and Z_IO_ and Z_IV_ denote the planting proportions of oats and common vetches, respectively, in the intercropping system. Oats are more competitive in the intercropping system when K_O_>K_V_, and common vetch are more competitive when K_O_<K_V_ (Litourgidis et al., 2011). Competitive ratio (CR) was used to assess the competitive ability of the component crops in an intercropping system (Dhima et al., 2007). It was calculated according to Equations 15, 16.


(15) $${\text{CR}}_{\text{O}}=\frac{{\text{LER}}_{\text{O}}}{{\text{LER}}_{\text{V}}}\times \frac{{\text{Z}}_{\text{IV}}}{{\text{Z}}_{\text{IO}}}$$

(16) $${\text{CR}}_{\text{V}}=\frac{{\text{LER}}_{\text{V}}}{{\text{LER}}_{\text{O}}}\times \frac{{\text{Z}}_{\text{IO}}}{{\text{Z}}_{\text{IV}}}$$

CR_O_ and CR_V_ represent the competition ratios of oats and common vetch, respectively. When CR_O_>1, oat is more competitive than common vetch in intercropping system; CR_O_<1, oat is less competitive than common vetch in intercropping system.

Actual yield loss index (AYL) indicates whether intercropped oats or intercropped common vetch lost or gained yield relative to monocropped oats or monocropped common vetch. (Equations 17–19) (Banik, 1996). As opposed to LER, AYL takes into consideration the actual sown proportion of land occupied by the component crops in the field.


(17) $$\text{AYL}={\text{AYL}}_{\text{O}}+{\text{AYL}}_{\text{V}}$$

(18) $${\text{AYL}}_{\text{O}}=\frac{{\text{Y}}_{\text{IO}}}{{\text{Z}}_{\text{IO}}}\times \frac{{\text{Z}}_{\text{MO}}}{{\text{Y}}_{\text{MO}}}–1$$

(19) $${\text{AYL}}_{\text{V}}=\frac{{\text{Y}}_{\text{IV}}}{{\text{Z}}_{\text{IV}}}\times \frac{{\text{Z}}_{\text{MV}}}{{\text{Y}}_{\text{MV}}}–1$$

Where AYL>0 indicates that the treatment exhibits gain compared to monoculture; AYL<0 indicates that the treatment exhibits loss compared to monoculture. the positive and negative of AYL_O_ and AYL_V_ indicate the contribution of oats or common vetch to the system indicating gain or loss. P_MO_ and P_MV_ represent the planting ratio of oats and common vetch in monoculture, respectively. Intercropping advantage (IA) was used to measure the economic viability of oats-common vetch intercropping systems (Banik, 1996). The index was derived from Equations 20–22.


(20) $$\text{IA}={\text{IA}}_{\text{O}}+{\text{IA}}_{\text{V}}$$

(21) $${\text{IA}}_{\text{O}}={\text{AYL}}_{\text{O}}\times {\text{P}}_{\text{O}}$$

(22) $${\text{IA}}_{\text{V}}={\text{AYL}}_{\text{V}}\times {\text{P}}_{\text{V}}$$

Aggressivity (A) was adopted as a competitive index to measure the extent at which the relative yield of one crop in the mixture was higher than that of the other, as expressed in Equations 23, 24) (Gitari et al., 2019).


(23) $${\text{A}}_{\text{O}}=\frac{{\text{Y}}_{\text{IO}}}{{\text{Y}}_{\text{MO}}\times {\text{Z}}_{\text{IO}}}-\frac{{\text{Y}}_{\text{IV}}}{{\text{Y}}_{\text{MV}}\times {\text{Z}}_{\text{IV}}}$$

(24) $${\text{A}}_{\text{V}}=\frac{{\text{Y}}_{\text{IV}}}{{\text{Y}}_{\text{MV}}\times {\text{Z}}_{\text{IV}}}-\frac{{\text{Y}}_{\text{IO}}}{{\text{Y}}_{\text{MO}}\times {\text{Z}}_{\text{IO}}}$$

#### 2.3.4 Statistical analysis

All the data were sequentially collected and sorted out through Microsoft 365 software (Microsoft, Inc., Redmond, Washington, USA). The statistical software SAS version 9.4 (SAS Institute Inc., Cary, North Carolina, USA) was applied for data variance analysis. One-way ANOVA was used to compare the differences between treatments in same year. Two-way ANOVA was used to compare the effects of individual factors and interactions between factors on the indicators. Year, cropping pattern, and sowing density were considered fixed effects, and replication was considered a random effect. Multiple comparisons of means among treatments were conducted by Fisher’s least significant difference (LSD) test at P< 0.05.

## 3 Results

### 3.1 Effect of sowing density and intercropping on grain yield

As shown in **Table 1**, the cropping pattern and sowing density had a significant effect on oat yield in both two years. Intercropping enhanced the oat grain yield by 23.12% to 30.98% across different sowing density, while reducing common vetch grain yield by 1.51% to 13.77%. With an increase in the oat sowing density, grain yield in both intercropping and monoculture exhibited an initial increase, followed by a subsequent decline. In the intercropping model, treatments with ID2, ID3, and ID4 consistently exhibited significantly higher oat grain yield than ID1 over the two years. Conversely, in the monoculture model, the SD2 and SD3 treatments showed a consistent and significant increase over SD1 for two years. Under intercropping conditions, oat yield reached its maximum in ID3 treatment, surpassing ID1 by significant margins of 12.81% and 16.59%. In the monoculture model, oat yield peaked at SD2 treatment, showed increases of 8.40% and 11.37% over SD1 in the two consecutive years. This suggests that intercropped oats can accommodate high planting densities. As the oat sowing density increased, the common vetch yield reached its maximum at the ID2 treatment over two years. Specifically, by 2022, the yield was significantly higher (8.16% compared with the ID1 treatment).

**Table 1:** Grain yield (kg ha^-1^) of oat and common vetch of different treatments in 2022 and 2023.

Treatment	2022		2023	
	Oat	Common vetch	Oat	Common vetch
ID1	3637.14 ± 94.83c	600.74 ± 25.44c	3375.24 ± 151.42c	616.78 ± 13.31bc
ID2	3968.29 ± 160.55ab	649.75 ± 26.31ab	3901.60 ± 108.48a	672.10 ± 44.32ab
ID3	4103.01 ± 68.55a	612.41 ± 42.19bc	3935.32 ± 110.96a	639.10 ± 35.96abc
ID4	3950.53 ± 114.42ab	606.81 ± 9.48bc	3685.06 ± 188.09b	616.10 ± 56.25bc
ID5	3783.21 ± 186.51bc	581.23 ± 10.03c	3472.94 ± 98.73c	588.44 ± 21.67c
SD1	2895.22 ± 91.60e	–	2741.5 ± 50.51f	–
SD2	3138.50 ± 93.48d	–	3053.19 ± 46.24d	–
SD3	3132.44 ± 86.07d	–	2984.80 ± 39.97de	–
SD4	3050.59 ± 54.94de	–	2872.47 ± 33.42ef	–
SD5	2955.60 ± 74.34de	–	2751.54 ± 85.49f	–
SV	–	668.17 ± 14.29a	–	682.39 ± 19.22a
Source of variance				
*P _Cropping pattern_*	**	–	**	–
*P _Sowing density_*	**	–	**	–
*P _CP_*_×_ *_SD_*	ns	–	ns	–

Values represent means ± SD. Different lowercase letters represented significant difference at p<0.05, the *, ** and ns. indicated significant levels at p< 0.05, p< 0.01 and p ≥ 0.05, respectively. S, sole cropping; I, oat-common vetch intercropping; V, common vetch; D1, D2, D3, D4 and D5 refer to oat sowing density of 4.50×10^6^ plants ha^-1^, 5.40×10^6^ plants ha^-1^, 6.30×10^6^ plants ha^-1^, 7.20×10^6^ plants ha^-1^, and 8.10×10^6^ plants ha^-1^. Different lowercase letters represented significant difference at p<0.05, the ** and ns. indicated significant levels at p< 0.01 and p ≥ 0.05, respectively.

### 3.2 Biological efficiency of the oat-common vetch intercropping systems

**Figure 2:**
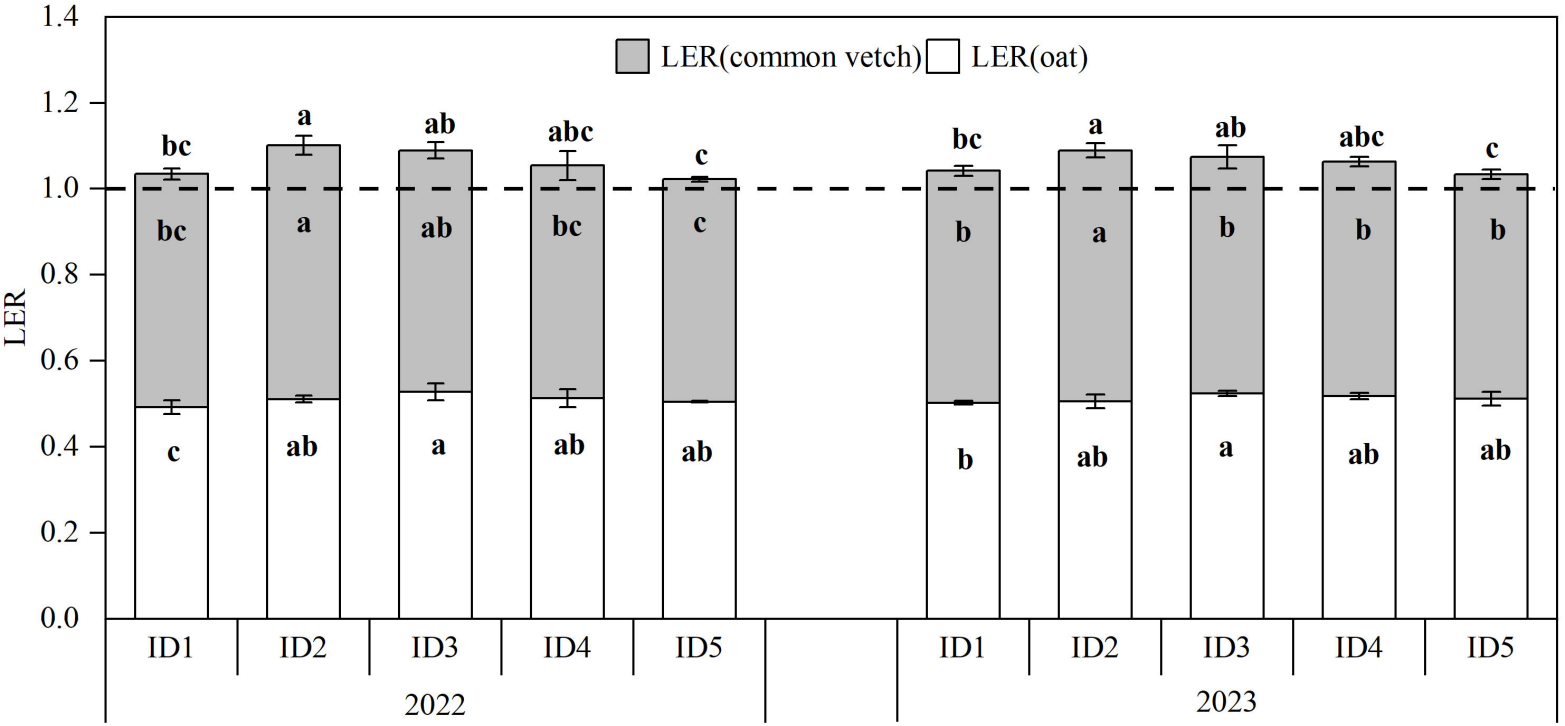
Land equivalent ratios (LERs) of oat-common vetch intercropping systems in 2022 and 2023. Bars bearing different letters within the same LERs indicate significance at p ≤ 0.05, different letters above the bars represent the significance of LER at p ≤ 0.05. Error bars epitomize the standard error of the means. The dashed lines denote an LER equal to 1. I, oat-common vetch intercropping; D1, D2, D3, D4 and D5 refer to oat sowing density of 4.50×10^6^ plants ha^-1^, 5.40×10^6^ plants ha^-1^, 6.30×10^6^ plants ha^-1^, 7.20×10^6^ plants ha^-1^, and 8.10×10^6^ plants ha^-1^.

The land equivalent ratio (LER) for the intercropping of oats with common vetch in grain production ranges from 1.03 to 1.09, indicating that this intercropping pattern can effectively enhance land utilization efficiency (**Figure 2**). The LER of the intercropping system showed a trend of an initial increase and subsequent decrease with increasing oat sowing density. The LER was maximized in both years under the ID2 treatment and was significantly higher than that under the ID1 and ID5 treatments. This suggested that increasing the density of the main crop improves intercropping productivity. However, excessive densification hindered further improvements in intercropping productivity. The LERo reached its maximum in both years under ID3 treatment, significantly increased by 4.27% to 7.19% compared to ID1, while LERv peaks under ID2 treatment, which significantly higher than that under ID1.

The land equivalent coefficient (LEC), system productivity index (SPI), and percentage yield difference (PYD) were significantly (P<0.01) influenced by oat sowing density, displaying an initial increase, followed by a decrease (**Table 2**). In terms of grain production, LEC, SPI, and PYD reached their maxima under the ID2 treatment and were significantly higher than the conventional density (ID1). The average SPI under the ID3 treatment was significantly increased by 13.04% compared to that under ID1 over two years, and the average PYD was significantly increased by 3.18% and 5.44%, respectively.

**Table 2:** Land equivalent coefficient (LEC), system productivity index (SPI) and percentage yield difference (PYD) as influenced by oat-common vetch intercropping systems.

Year	Treatment	LEC	SPI (kg ha ^-1^)	PYD (%)
2022	ID1	0.27bc	3017.20c	4.20c
	ID2	0.30a	3419.40a	8.93a
	ID3	0.29ab	3364.74a	7.38ab
	ID4	0.28abc	3242.87ab	6.31abc
	ID5	0.27c	3056.26bc	3.40c
2023	ID1	0.27bc	2836.92c	3.48c
	ID2	0.30a	3364.36a	10.16a
	ID3	0.30ab	3250.52ab	8.92ab
	ID4	0.28abc	3029.01bc	5.41bc
	ID5	0.26c	2812.83c	2.22c
Source of variance				
*P*	Year	ns	**	ns
	Treatment	**	**	**
	Year × Treatment	ns	ns	ns

Different lowercase letters represented significant difference at p<0.05, the ** and ns. indicated significant levels at p< 0.01 and p ≥ 0.05, respectively. I, oat-common vetch intercropping; D1, D2, D3, D4 and D5 refer to oat sowing density of 4.50×10^6^ plants ha^-1^, 5.40×10^6^ plants ha^-1^, 6.30×10^6^ plants ha^-1^, 7.20×10^6^ plants ha^-1^, and 8.10×10^6^ plants ha^-1^.

### 3.3 Economic efficiency of the intercropping systems

Calculating oat equivalent yield (OEY), monetary advantage index (MAI), relative value total (RVT), and replacement value of intercropping (RVI) provides an effective means of assessing the economic efficiency of increasing the intercropping sowing density (**Table 3**). The sowing density had a significant effect on economic efficiency indicators in both two years (P<0.01). With an increase in oat sowing density during intercropping, both OEY and MAI exhibited an initial increase, followed by a decrease. Elevating the oat sowing density enhanced the OEY of the intercropping system by 0.98%-13.90%. Notably, the ID2 and ID3 treatments showed significant average increases of 11.39% and 11.76%, respectively, compared to ID1. The MAI for each intercropping system was highest under the ID2 treatment, with a significant increase of 560.53 and 778.60 over ID1 in two years. However, MAI under the ID5 treatment was lower than that under ID1, indicating that excessive oat density increases the risk of system profit loss. Over the two years, RVT exhibited an initial increase followed by a decrease with changes in oat density, although the differences between treatments did not reach a significant level. RVI showed an increasing trend with an increase in oat density, with the ID5 treatment being significantly higher than ID1 over the two years, with an increase ranging from 6.08% to 7.45%.

**Table 3:** Oat equivalent yield (OEY), monetary advantage index (MAI), relative value total (RVT) and replacement value of intercropping (RVI) and as affected by oat-common vetch intercropping systems.

Year	Treatment	OEY	MAI	RVT	RVI
2022	ID1	1905.41c	461.76bc	0.65a	0.77c
	ID2	2074.62a	1022.29a	0.66a	0.77bc
	ID3	2100.51a	867.08ab	0.67a	0.79ab
	ID4	2035.31ab	723.77abc	0.67a	0.80a
	ID5	1949.20bc	385.64c	0.66a	0.81a
2023	ID1	1812.68b	366.45bc	0.66a	0.78b
	ID2	2064.72a	1145.05a	0.68a	0.79ab
	ID3	2053.45a	1009.32ab	0.69a	0.82ab
	ID4	1936.10ab	599.29abc	0.67a	0.82ab
	ID5	1830.51b	239.19c	0.67a	0.83a
Source of variance					
*P*	Year	*	ns	ns	*
	Treatment	**	**	ns	**
	Year × Treatment	ns	ns	ns	ns

Different lowercase letters represented significant difference at p<0.05, the *, ** and ns. indicated significant levels at p< 0.05, p< 0.01 and p ≥ 0.05, respectively. I, oat-common vetch intercropping; D1, D2, D3, D4 and D5 refer to oat sowing density of 4.50×10^6^ plants ha^-1^, 5.40×10^6^ plants ha^-1^, 6.30×10^6^ plants ha^-1^, 7.20×10^6^ plants ha^-1^, and 8.10×10^6^ plants ha^-1^.

### 3.4 Competition indices under the oat-common vetch intercropping systems

**Figure 3:**
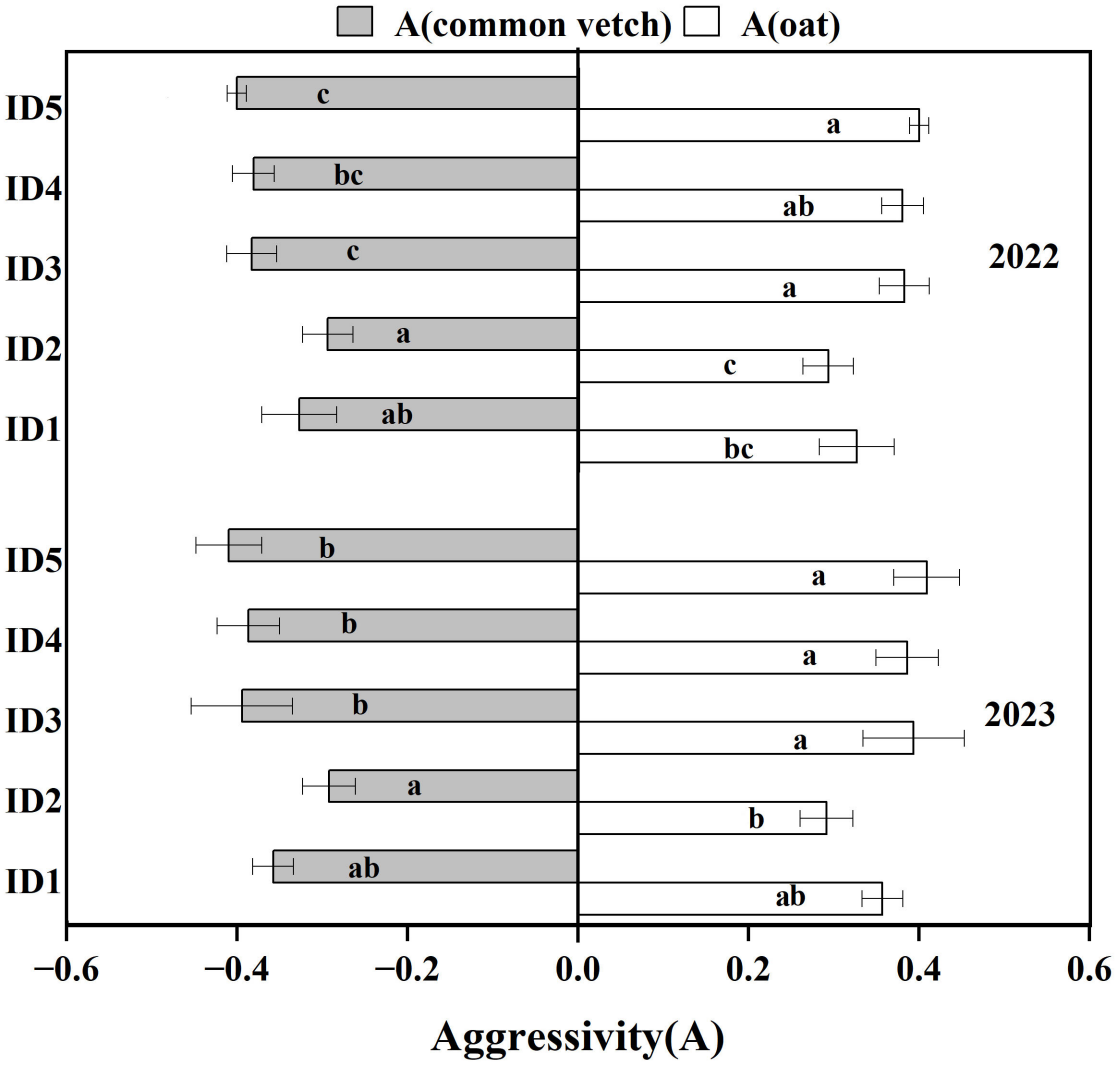
Aggressivity between oat and common vetch over 2022 and 2023 under intercropping systems. Bars bearing different letters within the same Aggressivity indicate significance at p ≤ 0.05. Error bars epitomize the standard error of the means. I, oat-common vetch intercropping; D1, D2, D3, D4 and D5 refer to oat sowing density of 4.50×10^6^ plants ha^-1^, 5.40×10^6^ plants ha^-1^, 6.30×10^6^ plants ha^-1^, 7.20×10^6^ plants ha^-1^, and 8.10×10^6^ plants ha^-1^.

In the two-year study, aggressivity of oats is consistently positive (0.29–0.41), while common vetch exhibits negative aggressivity values (-0.29–0.41), indicating that oats are the dominant crop in the intercropping system (**Figure 3**). Under different oat sowing densities, the Aggressivity of oats was the lowest in ID2, although the difference with ID1 was not significant. With an increase in ID3, ID4, and ID5, there was an increasing trend, with ID5 significantly increasing by 22.41% over ID1 in 2023. With the increase in oat sowing density, the actual yield losses of both oats and common vetch showed an initial increase, followed by a decrease (**Table 4**). Oats reached their maximum yield under ID3, and common vetches reached their maximum under the ID2 treatment, with both being significantly higher than that of ID1. The actual yield loss (AYL) of the intercropping system was highest under ID2, significantly exceeded ID1 and ID5. The results of intercropping advantage were similar to the actual yield losses, with IA_O_ being the highest under ID3, and IA_V_ and IA being the highest under ID2 and significantly exceeding ID1.

**Table 4:** Intercropping advantage (IA) and actual yield loss index (AYL) of oat-common vetch intercropping systems.

Year	Treatment	IA_O_	IA_V_	IA	AYLo	AYLv	AYL
2022	ID1	1.54b	-0.76b	0.78bc	0.26b	-0.10b	0.16b
	ID2	1.59ab	-0.21a	1.38a	0.26ab	-0.03a	0.24a
	ID3	1.86a	-0.63b	1.23ab	0.31a	-0.08b	0.23a
	ID4	1.77ab	-0.69b	1.08abc	0.29ab	-0.09b	0.20ab
	ID5	1.68ab	-0.97b	0.70c	0.28ab	-0.13b	0.15b
2023	ID1	1.39b	-0.72bc	0.67bc	0.23b	-0.10bc	0.14bc
	ID2	1.67ab	-0.12a	1.55a	0.28ab	-0.02a	0.26a
	ID3	1.91a	-0.48ab	1.43ab	0.32a	-0.06ab	0.25ab
	ID4	1.7ab	-0.74bc	0.96abc	0.28ab	-0.10bc	0.18abc
	ID5	1.57ab	-1.03c	0.54c	0.26ab	-0.14c	0.12c
Source of variance							
*P*	Year	ns	ns	ns	ns	ns	ns
	Treatment	*	**	**	*	**	**
	Year × Treatment	ns	ns	ns	ns	ns	ns

Different lowercase letters represented significant difference at p<0.05, the *, ** and ns. indicated significant levels at p< 0.05, p< 0.01 and p ≥ 0.05, respectively. I, oat-common vetch intercropping; D1, D2, D3, D4 and D5 refer to oat sowing density of 4.50×10^6^ plants ha^-1^, 5.40×10^6^ plants ha^-1^, 6.30×10^6^ plants ha^-1^, 7.20×10^6^ plants ha^-1^, and 8.10×10^6^ plants ha^-1^.

Under different oat sowing densities, the relative crowding coefficient of oats was highest in the ID3 treatment, significantly exceeding that of ID1 in both years (**Table 5**). Ko significantly increased by 19.76% and 21.94% over ID1 in two years, respectively. In the entire intercropping system, the relative crowding coefficient of ID2 was significantly higher than that of ID1 in both years. Among the increased density treatments, only ID5 decreases by 2.99–5.18% compared to ID1; however, the difference was not significant. The competition ratio for each treatment in both years showed that CR_O_>1 and CR_V_<1. Except for ID2, the competition ratio in each increased oat density treatment was higher than that in ID1, suggesting that increasing the density of the main crop could enhance the competitiveness of oats. The competition ratio of common vetch showed an initial increase followed by a decrease, indicating that appropriately increasing oat density is beneficial for improving intercropping competitiveness, but excessive density is detrimental to the increased production of common vetch.

**Table 5:** Relative crowding coefficient (K) and Competitive ratio (CR) of oat-common vetch intercropping systems.

Year	Treatment	K_O_	K_V_	K	CR_O_	CR_V_
2022	ID1	1.52b	0.78b	1.18bc	1.40ab	0.72b
	ID2	1.54ab	0.94a	1.44a	1.30b	0.77a
	ID3	1.65a	0.82b	1.35ab	1.43a	0.70b
	ID4	1.61ab	0.80b	1.29abc	1.43a	0.70b
	ID5	1.57ab	0.73b	1.15c	1.47a	0.68b
2023	ID1	1.46b	0.79bc	1.15bc	1.36bc	0.73ab
	ID2	1.57ab	0.96a	1.52a	1.30c	0.77a
	ID3	1.68a	0.86ab	1.44ab	1.41ab	0.71bc
	ID4	1.58ab	0.79bc	1.26abc	1.42ab	0.70bc
	ID5	1.53ab	0.71c	1.09c	1.46a	0.68c
Source of variance						
*P*	Treatment	ns	ns	ns	ns	ns
	Year	*	**	**	**	**
	Year × Treatment	ns	ns	ns	ns	ns

Different lowercase letters represented significant difference at p<0.05, the *, ** and ns. indicated significant levels at p< 0.05, p< 0.01 and p ≥ 0.05, respectively. I, oat-common vetch intercropping; D1, D2, D3, D4 and D5 refer to oat sowing density of 4.50×10^6^ plants ha^-1^, 5.40×10^6^ plants ha^-1^, 6.30×10^6^ plants ha^-1^, 7.20×10^6^ plants ha^-1^, and 8.10×10^6^ plants ha^-1^.

## 4 Discussion

Intercropping is recognized as an essential planting pattern in China because of its efficient utilization of light, heat, water, and nutrient resources through temporal niche separation and spatial niche complementarity (Li et al., 2014, 2023). The results from the two-year study indicate that intercropping significantly increased oat grain yield by 23.12–30.98% under different planting densities compared to monoculture, and demonstrated a consistent yield advantage, as observed in previous studies (Wang et al., 2020; Li et al., 2021). This also confirms the potential of oat and common vetch intercropping patterns for grain production. The primary reason for the yield advantage of intercropping lies in the combination of the fibrous root system of oats and the taproot system of common vetch in the cereal-legume intercropping system (Wang et al., 2018; Zhang et al., 2021; Peng et al., 2022). Additionally, the rational combination of tall-stem and short-stem crops in the aboveground portion enhances light capture by the leaves, increases canopy interception capacity, and promotes yield formation (Gou et al., 2017; Li et al., 2020; Lu et al., 2023). Increasing the sowing density to enhance the utilization efficiency of light and temperature resources and unlocking collective production potential are crucial for achieving high yield (Yang et al., 2022). In this experiment, as the oat sowing density increased, the grain yield in both the intercropping and monoculture systems exhibited a trend of initial increase, followed by a decrease. In the intercropping mode, oat yield reached its maximum under the ID3 treatment, which was significantly increased by 12.81% and 16.59% over ID1 in the two years, respectively. In the monoculture mode, the yield reached its maximum under SD2 density, with an increase of 8.40% and 11.37% over SD1 in the two years. Across all density treatments, the average yield increases for intercropping compared to monoculture were 24.37%, 27.11%, 31.41%, 28.89%, and 27.11%, indicating that intercropped oats had a greater yield advantage in the ID3 treatment. This suggests that intercropped oats can accommodate higher sowing densities than monocultures, which is consistent with previous research findings (Moreira et al., 2015).

Previous studies have shown that intercropping oats with common vetch significantly increase photosynthetically active radiation interception by oats, while decreasing interception by common vetch, thereby enhancing the overall light use efficiency of the intercropping system (Li et al., 2014). However, overcrowded plant populations exacerbate intraspecies competition, worsen canopy conditions, and reduce individual photosynthetic capacity, resulting in decreased grain yield per plant (Fan et al., 2022). Intercropping oats with common vetch reduced the common vetch grain yield by 1.51–13.77%, which indicating that the yield increase in oats comes at the cost of reduced common vetch yield. Compared to other intercropping systems, such as maize with peanuts (Wang et al., 2021 or corn intercropped with pea (Yang et al., 2021), the reduction in yield in the oat and common vetch intercropping systems is relatively small. This is likely because oats provide excellent support for the growth of common vetches, allowing them to capture more light by increasing plant height and overcoming competition from oats (Li et al., 2020). With an increase in oat sowing density, the common vetch yield reached its maximum with the ID2 treatment in both years, which had a significant increase of 8.16% over ID1 in 2022. This suggests that moderately increasing oat density is beneficial for increasing common vetch yield, possibly by alleviating nitrogen constraints imposed by oats (Neugschwandtner and Kaul, 2015).

Interplanting crops exhibits both competition and complementarity when utilizing space and various resources. When complementarity between species outweighs competition, the intercropping system enhances resource utilization, demonstrates intercropping superiority, and promotes collective productivity (Ren et al., 2016). Effective intercropping coordination enhances crop yield and land-use efficiency, which is primarily reflected in the LER (Raza et al., 2019). In the present study, the average LER of oats intercropped with common vetch was 1.06, indicating a grain-yield advantage in this intercropping model. In comparison, monoculture would require a 6% increase in land area to achieve equivalent production (Yilmaz et al., 2015). The LER in the intercropping system increased with a moderate increase in the oat sowing density. The LER was highest in the ID2 treatment for both years and significantly surpassed the ID1 and ID5 treatments. Cereal crops generally outcompete legumes for soil mineral nitrogen, forcing them to rely more on nitrogen fixation, while also facilitating nitrogen fixation in legumes and the subsequent transfer of nitrogen to non-leguminous crops (Gao et al., 2022). The increase in oat sowing density contributes to enhanced LER, which may be attributed to the intercropping system alleviating nitrogen inhibition by common vetch (Monti et al., 2016). However, as the oat sowing density continued to increase, the LER of the intercropping system decreased. The ID3, ID4, and ID5 treatments showed average LER reductions of 1.28%, 3.36%, and 6.14%, respectively. Compared to the ID2 treatment, the ID5 treatment consistently significantly lower than that of the ID2 treatment over two years. This decrease may be associated with the intensified competition between among plants for water, light, and nutrients, resulting in reduced accessibility to assimilable light (Moreira et al., 2015). LERo was the highest in the ID3 treatment for both years, showing a significant increase of 4.27% to 7.19% compared with the ID1 treatment. LERv peaked in the ID2 treatment and significantly surpassed that in the ID1 treatment. These findings suggest that increasing the oat sowing density can influence common vetch land-use efficiency and highlight the synergistic relationship between the two crops. In all density treatments, the LEC exceeded 0.25 (Jardim et al., 2021), the SPI surpassed that of monoculture oats, and the PYD was consistently positive. All biological indicators reached their maximum values in the ID2 treatment, which emphasized that a moderate increase in the oat sowing density enhanced the biological efficiency of the intercropping system.

Because more than one crop is involved in intercropping systems, which typically have different selling prices and production costs, various indices can be employed to assess the economic efficiency of such systems (Machiani et al., 2018; Soratto et al., 2022). In this study, the OEY over two years was lower than that of monoculture oats, and the RVT was consistently less than 1. This outcome primarily stems from the considerably lower grain yield per unit area of common vetch than that compared of oats, despite its price being only 25% higher (Verma et al., 2013). Increasing the oat sowing density enhanced the OEY of the intercropping system, with significant improvements of 11.39% and 11.76% in the ID2 and ID3 treatments, respectively. The MAI is positive in all scenarios, indicating that the oats-common vetch intercropping model is more profitable than the monoculture approach (Soratto et al., 2022). As the oat sowing density increased, the MAI initially increased and then declined, suggesting that a moderate increase in oat density during intercropping could lead to higher profitability. Similar conclusions were drawn for the RVI. The IA represents the economic superiority of the intercropping system (Machiani et al., 2018), with IA_O_ and IA values exceeding 1 for all treatments, indicating that the intercropping model outperformed the monoculture in terms of profitability. However, IA_V_ values less than 1 in each treatment in the two years could explain economic disadvantage observed in the OEY, RVT, and RVI values.

The relative crowding coefficient (K) measured the competitive advantage of one crop over another in an intercropping system. In this study, K_O_ values at all densities were greater than K_V_ values, indicating that oats were the dominant crop in the intercropping system (Abou El-Enin et al., 2023). The K values for the intercropping system are greater than 1 in both years. This indicated that intercropping systems exhibit less interspecific competition compared to intraspecific competition in monoculture, signifying a productivity advantage of intercropping (Banik, 1996). At different oat sowing densities, the K value of the ID2 treatment was the highest, indicating that the ID2 treatment can effectively coordinate the interspecific relationship between the two crops. K_O_ and K_V_ reached their maximum values under the ID3 and ID2 treatments, respectively. This also suggests that optimizing the intraspecific competition of common vetch is an important direction for further enhancing the productivity of the intercropping system. In all treatments, the competition ratio between oat and common vetch was positive, with CR_O_ being greater than CR_V_, indicating that oats were more competitive than common vetch and were the dominant intercropping crop. This is consistent with the results of a previous study of Li et al. (2020). The competition ratio (CR_V_) for intercropped common vetch was positive, indicating the feasibility of intercropping at different oat planting densities. Among all density treatments, CR_O_ was the smallest and CR_V_ reached the largest in the ID2 treatment, it indicated that ID2 density could coordinate competition between intercropped crops to achieve synergistic yield increases, a trend also reflected in the Aggressivity.

Actual yield loss (AYL) represents the relative yield loss or gain in intercropping systems. In the present study, AYL was greater than 0, indicating intercropping superiority, and AYL in the ID2 treatment is significantly higher than that in the ID1 treatment, suggesting that moderate densification could enhance the efficiency of intercropping systems. In the present study, AYL_O_ was maximized at the ID3 density, whereas AYL_V_ reached maximized at the ID2 density. The phenomenon could be attributed to oats increasing their competitiveness with rising crop density to ensure resource acquisition for high-density populations, while excessive competition may impede the yield of common vetch (Neugschwandtner and Kaul, 2015). The positive values of AYL_O_ for all treatments indicate that oats exhibit a yield advantage in the intercropping system, which is consistent with the result that oats are the competitive dominant crop and common vetch is the competitive inferior crop in the intercropping system (Li et al., 2020).

## 5 Conclusion

Our study showed a significant advantage in grain yield production for the oat-common vetch intercropping model compared with sole cropping, demonstrating higher adaptability to increased sowing densities. With a 20% increase in the oat sowing density, both oat and common vetch yields were significant improvement. Moderate increases in sowing density also contributed to the economic benefits of the intercropping system. In the intercropping system, oats exhibited a competitive advantage. The moderate increase in sowing density primarily alleviated the interspecific competition between oats and common vetches, synergistically enhanced the overall yield. This study provides novel insights into the grain production of oats and common vetch in arid and semi-arid regions. In further investigations, we should pay more attention to optimizing crop spatial arrangement and nitrogen usage to determine if it can promote intercropping systems to accommodate larger densities, thereby achieving higher levels of grain yield.

## Data availability statement

The datasets presented in this article are not readily available because no limite. Requests to access the datasets should be directed to Ying Wang 1269046052@qq.com.

## Author contributions

YW: Conceptualization, Investigation, Writing – original draft, Writing – review & editing. XH: Writing – review & editing. XZ: Writing – review & editing. YZ: Data curation, Methodology, Software, Writing – original draft. BQ: Conceptualization, Investigation, Methodology, Writing – original draft, Writing – review & editing. LL: Writing – review & editing, Conceptualization, Methodology, Supervision, Project Administration, Funding Acquisition.

## References

[B1] Abdul Rahman N. Larbi A. Kotu B. Asante M. O. Akakpo D. B. Mellon-Bedi S.. (2021). Maize–legume strip cropping effect on productivity, income, and income risk of farmers in northern Ghana. Agron. J. 113, 1574–1585. doi: 10.1002/agj2.20536

[B2] Abou El-Enin M. M. Sheha A. M. El-Serafy R. S. Ali O. A. Saudy H. S. Shaaban A. (2023). Foliage-sprayed nano-chitosan-loaded nitrogen boosts yield potentials, competitive ability, and profitability of intercropped maize-soybean. Int. J. Plant Prod. 17, 517–542. doi: 10.1007/s42106-023-00253-4

[B3] Adetiloye P. O. Ezedinma F. O. C. Okigbo B. N. (1983). A land equivalent coefficient (LEC) concept for the evaluation of competitive and productive interactions in simple to complex crop mixtures. Ecol. Model. 19, 27–39. doi: 10.1016/0304-3800(83)90068-6

[B4] Afe A. Atanda S. (2015). Percentage yield difference, an index for evaluating intercropping efficiency. Am. J. Exp. Agric. 5, 459–465. doi: 10.9734/AJEA/2015/12405

[B5] Alabi A. Esobhawan O. (2006). Relative economic value of maize-okra intercrops in rainforest zone, *Nigeria* . J. Cent. Eur. Agric. 7, 875–879. doi: 10.5513/JCEA.V7I3.393

[B6] Banik P. (1996). Evaluation of wheat (*Triticum aestivum*) and legume intercropping under 1: 1 and 2: 1 Row-replacement series system. J. Agron. Crop Sci. 176, 289–294. doi: 10.1111/J.1439-037X.1996.TB00473.X

[B7] Dhima K. V. Lithourgidi A. S. Vasilakoglou I. B. Dordas C. A. (2007). Competition indices of common vetch and cereal intercrops in two seeding ratio. Field Crops Res. 100, 249–256. doi: 10.1016/j.fcr.2006.07.008

[B8] Fan H. Yin W. Zhao C. Yu A. Z. Fan Z. L. Hu F. L.. (2022). Photophysiological mechanism of dense planting to increase the grain yield of intercropped maize with nitrogen-reduction application in arid conditions. Agronomy 12, 2994. doi: 10.3390/agronomy12122994

[B9] Fan Z. L. Zhao Y. H. Chai Q. Zhao C. Yu A. Z. Coulter J. A.. (2019). Synchrony of nitrogen supply and crop demand are driven via high maize density in maize/pea strip intercropping. Sci. Rep. 9, 10954. doi: 10.1038/s41598-019-47554-1 31358903 PMC6662835

[B10] Finney D. (1990). Intercropping experiments, statistical analysis, and agricultural practice. Exp. Agric. 26, 73–81. doi: 10.1017/S0014479700015428

[B11] Freschet G. T. Violle C. Bourget M. Y. Schere-Lorenzen M. Fort F. (2018). Allocation, morphology, physiology, architecture: The multiple facets of plant above-and below-ground responses to resource stress. New Phytol. 219, 1338–1352. doi: 10.1111/nph.15225 29856482

[B12] Gao H. X. Zhang C. C. van der Werf W. Ning P. Zhang Z. Wan S. B.. (2022). Intercropping modulates the accumulation and translocation of dry matter and nitrogen in maize and peanut. Field Crops Res. 284, 108561. doi: 10.1016/j.fcr.2022.108561

[B13] Ghosh P. K. (2004). Growth, yield, competition and economics of groundnut/cereal fodder intercropping systems in the semi-arid tropics of India. Field Crops Res. 88, 227–237. doi: 10.1016/j.fcr.2004.01.015

[B14] Gitari H. I. Nyawade S. O. Kamau S. Gachenf C. K. K. Karanja N. N. Schulte-Geldermann E. (2019). Increasing potato equivalent yield increases returns to investment under potato-legume intercropping systems. Open Agric. 4, 623–629.9. doi: 10.1515/opag-2019-0062

[B15] Gitari H. I. Nyawade S. O. Kamau S. Karanja N. N. Gachene C. K. K. Raza M. A.. (2020). Revisiting intercropping indices with respect to potato-legume intercropping systems. Field Crops Res. 258, 107957. doi: 10.1016/j.fcr.2020.107957

[B16] Gong X. W. Ferdinand U. Dang K. Li J. Chen G. H. Luo Y.. (2020). Boosting proso millet yield by altering canopy light distribution in proso millet/mung bean intercropping systems. Crop J. 8, 365–377. doi: 10.1016/j.cj.2019.09.009

[B17] Gou F. Van Ittersum M. K. Simon E. Leffelaar P. A. van der Putten P. E. Zhang L. Z.. (2017). Intercropping wheat and maize increases total radiation interception and wheat RUE but lowers maize RUE. Eur. J. Agron. 84, 125–139. doi: 10.1016/j.eja.2016.10.014

[B18] Han F. Guo S. Q. Wei S. Guo R. Cai T. Zhang P.. (2022). Photosynthetic and yield responses of rotating planting strips and reducing nitrogen fertilizer application in maize–peanut intercropping in dry farming areas. Front. Plant Sci. 13. doi: 10.3389/fpls.2022.1014631 PMC9708908 36466232

[B19] Hauggaaed-Nielsen H. Andersen M. K. Joernsgaard B. Jensen E. S. (2006). Density and relative frequency effects on competitive interactions and resource use in pea–barley intercrops. Field Crops Res. 95, 256–267. doi: 10.1016/j.fcr.2005.03.003

[B20] Jardim A. M. D. R. F. Da Silva T. G. F. De Souza L. S. B. Júnior G. D. N. A. Alves H. K. M. N. DE Sá Souza M.. (2021). Intercropping forage cactus and sorghum in a semi-arid environment improves biological efficiency and competitive ability through interspecific complementarity. J. Arid Environ. 188, 104464. doi: 10.1016/j.jaridenv.2021.104464

[B21] Jiang S. C. Du B. Wu Q. X. Zhang H. W. Zhu J. Q. (2023). Increasing pit-planting density of rice varieties with different panicle types to improves sink characteristics and rice yield under alternate wetting and drying irrigation. Food Energy Secur. 12, e335. doi: 10.1002/fes3.335

[B22] Li C. J. Syomoh T. J. Makowski D. Li H. G. Zhang C. C. Zhang F. S.. (2023). The productive performance of intercropping. Proc. Natl. Acad. Sci. 120, e2201886120. doi: 10.1073/pnas.2201886120 36595678 PMC9926256

[B23] Li L. Tilman D. Lambers H. Zhang F. S. (2014). Plant diversity and overyielding: insights from belowground facilitation of intercropping in agriculture. New Phytol. 203, 63–69. doi: 10.1111/nph.12778 25013876

[B24] Li R. Zhang Z. X. Tang W. Huang Y. F. Coulter J. A. Nan Z. B. (2020). Common vetch cultivars improve yield of oat row intercropping on the Qinghai-Tibetan plateau by optimizing photosynthetic performance. Eur. J. Agron. 117, 126088. doi: 10.1016/j.eja.2020.126088

[B25] Li R. Zhang Z. X. Tang W. Huang Y. F. Nan Z. B. (2021). Effect of row configuration on yield and radiation use of common vetch-oat strip intercropping on the Qinghai-Tibetan plateau. Eur. J. Agron. 128, 126290. doi: 10.1016/j.eja.2021.126290

[B26] Li Y. L. Cui J. Y. Zhang T. H. Zhao H. L. (2003). Measurement of evapotranspiration of irrigated spring wheat and maize in a semi-arid region of north China. Agric. Water Manage. 61, 1–12. doi: 10.1016/S0378-3774(02)00177-4

[B27] Lithourgidis A. Vasilakoglou I. Dhima K. Dordas C. Yiakoulaki M. (2006). Forage yield and quality of common vetch mixtures with oat and triticale in two seeding ratios. Field Crops Res. 99, 106–113. doi: 10.1016/j.fcr.2006.03.008

[B28] Litourgidis A. Vlachostergios D. Dordas C. Damalas C. (2011). Dry matter yield, nitrogen content, and competition in pea–cereal intercropping systems. Eur. J. Agron. 34, 287–294. doi: 10.1016/j.eja.2011.02.007

[B29] Lu J. T. Dong Q. Q. Lan G. H. He Z. C. Zhou D. Y. Zhang H.. (2023). Row ratio increasing improved light distribution, photosynthetic characteristics, and yield of peanut in the maize and peanut strip intercropping system. Front. Plant Sci. 14. doi: 10.3389/fpls.2023.1135580 PMC10377676 37521911

[B30] Machiani M. A. Javamard A. Morshedloo M. R. Maggi F. (2018). Evaluation of competition, essential oil quality and quantity of peppermint intercropped with soybean. Ind. Crops Prod. 111, 743–754. doi: 10.1016/j.indcrop.2017.11.052

[B31] Maingi J. M. Shisanya C. A. Gitonga N. M. Hornetz B. (2001). Nitrogen fixation by common bean (Phaseolus vulgaris L.) in pure and mixed stands in semi-arid south-east Kenya. Eur. J. Agron. 14, 1–12. doi: 10.1016/S1161-0301(00)00080-0

[B32] Mead R. Willey R. W. (1980). The concept of a ‘Land equivalent ratio’ and advantages in yields from intercropping. Exp. Agric. 16, 217–228. doi: 10.1017/S0014479700010978

[B33] Monti M. Pellicanò A. Santonoceto C. Preiti G. Pristeri A. (2016). Yield components and nitrogen use in cereal-pea intercrops in Mediterranean environment. Field Crops Res. 196, 379–388. doi: 10.1016/j.fcr.2016.07.017

[B34] Moreira A. Moraes L. A. Schroth G. Mandarino J. M. (2015). Effect of nitrogen, row spacing, and plant density on yield, yield components, and plant physiology in soybean–wheat intercropping. Agron. J. 107, 2162–2170. doi: 10.2134/agronj15.0121

[B35] Moseley W. (1994). An equation for the replacement value of agroforestry. Agroforestry Syst. 26, 47–52. doi: 10.1007/BF00705151

[B36] Neugschwandtner R. W. Kaul H. P. (2015). Nitrogen uptake, use and utilization efficiency by oat–pea intercrops. Field Crops Res. 179, 113–119. doi: 10.1016/j.fcr.2015.04.018

[B37] Nguyen V. Riley S. Nagel S. Fisk I. Searle I. R. (2020). Common vetch: a drought tolerant, high protein neglected leguminous crop with potential as a sustainable food source. Front. Plant Sci. 11. doi: 10.3389/fpls.2020.00818 PMC7316993 32636858

[B38] Odo P. E. (1991). Evaluation of short and tall sorghum varieties in mixtures with cowpea in the Sudan savanna Of Nigeria: land equivalent ratio, grain yield and system productivity index. Exp. Agric. 27, 435–441. doi: 10.1017/S0014479700019426

[B39] Peng Z. Guo X. Z. Xiang Z. X. Liu D. H. Yu K. Sun K.. (2022). Maize intercropping enriches plant growth-promoting rhizobacteria and promotes both the growth and volatile oil concentration of Atractylodes lancea. Front. Plant Sci. 13. doi: 10.3389/fpls.2022.1029722 PMC9638049 36352878

[B40] Raza M. A. Feng L. Y. van der Werf W. Cai G. R. Khalid M. H. B. Iqbal N.. (2019). Narrow-wide-row planting pattern increases the radiation use efficiency and seed yield of intercrop species in relay-intercropping system. Food Energy Secur. 8, e170. doi: 10.1002/fes3.170 PMC6391028 30807607

[B41] Ren Y. Y. Liu J. J. Wang Z. L. Zhang S. Q. (2016). Planting density and sowing proportions of maize–soybean intercrops affected competitive interactions and water-use efficiencies on the Loess Plateau, China. European. J. Agron. 72, 70–79. doi: 10.1016/j.eja.2015.10.001

[B42] Singh R. Ahlawat I. Sharma N. (2015). Resource use efficiency of transgenic cotton and peanut intercropping system using modified fertilization technique. Int. J. Plant Prod. 9, 523–540. doi: 10.22069/IJPP.2015.2461

[B43] Soratto R. P. Perdoná M. J. Parecido R. J. Pinotti R. N. Gitari H. I. (2022). Turning biennial into biannual harvest: Long-term assessment of Arabica coffee–macadamia intercropping and irrigation synergism by biological and economic indices. Food Energy Secur. 11, e365. doi: 10.1002/fes3.365

[B44] Verma R. K. Chauhan A. Verma R. S. Rahman L. U. Bisht A. (2013). Improving production potential and resources use efficiency of peppermint (*Mentha piperita* L.) intercropped with geranium (*Pelargonium graveolens* L. Herit ex Ait) under different plant density. Ind. Crops Prod. 44, 577–582. doi: 10.1016/j.indcrop.2012.09.019

[B45] Wang Q. Bai W. Sun Z. X. Zhang D. S. Zhang Y. Wang R. N.. (2021). Does reduced intraspecific competition of the dominant species in intercrops allow for a higher population density? Food Energy Secur. 10, 285–298. doi: 10.1002/fes3.270

[B46] Wang Y. F. Qin Y. Z. Chai Q. Feng F. X. Zhao C. Yu A. Z. (2018). Interspecies interactions in relation to root distribution across the rooting profile in wheat-maize intercropping under different plant densities. Front. Plant Sci. 9. doi: 10.3389/fpls.2018.00483 PMC5920641 29731758

[B47] Wang Z. K. Jiang H. L. Shen Y. Y. (2020). Forage production and soil water balance in oat and common vetch sole crops and intercrops cultivated in the summer-autumn fallow season on the Chinese Loess Plateau. Eur. J. Agron. 115, 126042. doi: 10.1016/j.eja.2020.126042

[B48] Willey R. Osiru D. (1972). Studies on mixtures of maize and beans (*Phaseolus vulgaris*) with particular reference to plant population. J. Agric. Sci. 79, 517–529. doi: 10.1017/S0021859600025909

[B49] Xu R. X. Zhao H. M. Liu G. B. You Y. L. Ma L. Liu N.. (2021). Effects of nitrogen and maize plant density on forage yield and nitrogen uptake in an alfalfa–silage maize relay intercropping system in the North China Plain. Field Crops Res. 263, 108068. doi: 10.1016/j.fcr.2021.108068

[B50] Yang H. W. Chai Q. Yin W. Hu F. L. Qin A. Z. Fan Z. L.. (2022). Yield photosynthesis and leaf anatomy of maize in inter-and mono-cropping systems at varying plant densities. Crop J. 10, 893–903. doi: 10.1016/j.cj.2021.09.010

[B51] Yang H. W. Hu F. L. Yin W. Chai Q. Zhao C. Yu A. Z.. (2021). Integration of tillage and planting density improves crop production and carbon mitigation of maize/pea intercropping in the oasis irrigation area of northwestern China. Field Crops Res. 272, 108281. doi: 10.1016/j.fcr.2021.108281

[B52] Yilmaz Ş. Özel A. Atak M. Erayman M. (2015). Effects of seeding rates on competition indices of barley and vetch intercropping systems in the Eastern Mediterranean. Turkish J. Agric. Forest. 39, 135–143. doi: 10.3906/tar-1406-155

[B53] Yu Y. Stomph T. J. Makowski D. van der Werf W. (2015). Temporal niche differentiation increases the land equivalent ratio of annual intercrops: a meta-analysis. Field Crops Res. 184, 133–144. doi: 10.1016/j.fcr.2015.09.010

[B54] Zhai L. C. Xie R. Z. Bo M. Li S. K. (2018). Evaluation and analysis of intraspecific competition in maize: A case study on plant density experiment. J. Integr. Agric. 17, 2235–2244. doi: 10.1016/S2095-3119(18)61917-3

[B55] Zhang D. S. Sun Z. X. Feng L. S. Bai W. Yang N. Zhang Z.. (2020). Maize plant density affects yield, growth and source-sink relationship of crops in maize/peanut intercropping. Field Crops Res. 257, 107926. doi: 10.1016/j.fcr.2020.107926

[B56] Zhang W. Li S. Q. Shen Y. F. Yue S. C. (2021). Film mulching affects root growth and function in dryland maize-soybean intercropping. Field Crops Res. 271, 108240. doi: 10.1016/j.fcr.2021.108240

